# Effectiveness of smoking cessation interventions in pregnant women attending primary care: a scoping review

**DOI:** 10.3399/BJGPO.2023.0185

**Published:** 2024-07-24

**Authors:** Niamh Connolly, Dervla Kelly, Patrick O'Donnell, Sarah Hyde

**Affiliations:** 1 School of Medicine, University of Limerick, Limerick, Ireland

**Keywords:** smoking intervention, smoking, pregnancy, primary health care

## Abstract

**Background:**

Smoking during pregnancy has many adverse effects for infant and mother. Despite this, many pregnant women continue smoking. Primary care is a suitable area to provide smoking cessation interventions.

**Aim:**

To investigate available literature regarding effectiveness of smoking cessation interventions for pregnant women in primary care, the factors contributing to this effectiveness, and to provide suggestions for future research.

**Design & setting:**

Systematic scoping literature review.

**Method:**

The methodology followed Preferred Reporting Items for Systematic Reviews and Meta-Analyses (PRISMA) extension for scoping reviews. Five electronic databases were searched. Inclusion criteria included original research studies and studies published in English. Data were extracted using a modified Joanna Briggs Institute (JBI) data-charting tool.

**Results:**

The initial search yielded 878 articles. Following article screening, 12 studies were included. Five studies found a statistically significant increase in smoking cessation rates or reduction in tobacco consumed in the intervention group. The remaining studies showed no significant difference between the groups. However, 10 studies showed the control group received usual antenatal care involving smoking cessation promotion. An increase in smoking cessation rates was seen in intervention and control groups, demonstrating the effectiveness of these interventions. Interventions included education, counselling, self-help, and financial incentives. They were delivered by GPs, midwives, counsellors, and pregnancy advisers.

**Conclusion:**

Primary care is suitable to offer smoking cessation interventions to pregnant women, as it is often the first point of care and more easily accessible than secondary care. Future research is needed to determine the most effective types of interventions.

## How this fits in

The World Health Organization (WHO) recommends that all pregnant women who use tobacco should be offered a brief intervention, despite the quality of evidence for this being low. This review shows that primary care is a suitable location to provide this service. However, further research is needed to determine if reduction in tobacco consumption is owing to interventions, or whether pregnancy itself and/or increased contact with healthcare professionals are the main motivators for behavioural change. There is scope for future research to identify the most effective smoking cessation interventions and how they can be delivered.

## Introduction

Tobacco use during pregnancy has many negative outcomes, including increased risk of miscarriage, stillbirth, and premature labour.^1^ The Centers for Disease Control and Prevention (CDC) report that 20% of babies born to mothers who smoke are of low birth weight and more likely to die from sudden infant death syndrome.^2^ The infant mortality rate in those born to mothers who smoke is estimated to be 40% higher than infants not exposed to tobacco.^3^ Smoking in pregnancy is associated with increased obstetric risk^4–6^ and linked with adverse effects to the baby later in life, including increased risk of asthma,^7^ obesity,^8^ and reduced academic achievement.^9^

Unfortunately, many pregnant women continue to smoke. A 2018 meta-analysis reported that 8% of women in Europe smoked during pregnancy.^10^ In the US, 7.2% of women who gave birth in 2016 smoked during pregnancy.^11^ This behaviour causes financial burdens on healthcare systems. A study in England showed that children of women who smoked during pregnancy have higher healthcare costs during their first 5 years of life.^12^ The quantity of cigarettes influences the financial impact, with children of mothers smoking >20 cigarettes daily having the highest cost difference.^13^ In 1993, tobacco use during pregnancy resulted in healthcare costs of $135–$167 million in the US.^14^

The World Health Organization (WHO) states that primary care is an effective healthcare setting for providing smoking cessation support to all patients.^15^ It is recommended that pregnant smokers should be offered advice, behavioural support, and pharmacotherapy.^16^ The American College of Obstetricians and Gynecologists (ACOG) suggests that primary care professionals should provide interventions during pregnancy.^17^ However, it has been reported that community healthcare providers miss opportunities for interventions by not discussing tobacco use consistently with patients who are pregnant.^18^

Several smoking cessation interventions during pregnancy have been studied. For motivational interviewing, results are mixed with some studies showing increased cessation rates and others demonstrating no improvement.^19,20^ Nicotine replacement therapy (NRT) effects in pregnancy are also mixed.^21–23^ A Cochrane review stated further research is needed on the efficacy and safety of pharmacotherapy for smoking cessation in pregnancy.^24^

Opinions of primary care professionals and patients regarding provision of smoking cessation services have been investigated. Obstacles described include lack of resources, time, training and clarity regarding policies and guidelines.^25–28^ One review identified that although primary care professionals accept their role in promoting smoking cessation, they disagree on the level of involvement they should have.^27^ Patients believe that smoking cessation should be discussed routinely during general practice consultations.^29^

Evidence regarding smoking cessation services for pregnant women in primary care is lacking. The aim of this review is to assess the effectiveness of smoking cessation interventions in pregnant women in primary care. A scoping review was done to map existing literature and identify knowledge gaps. To our knowledge, this is the first scoping review to do so. Mapping existing literature will contribute to developing effective smoking cessation strategies for pregnant women. This can allow a more specific research question to be answered by a systematic or meta-analysis study.

## Method

A systematic scoping review was completed, adhering to the Preferred Reporting Items for Systematic Reviews and Meta-Analyses (PRISMA) extension for scoping reviews.^30^ The methodology was based on Arksey and O’Malley’s framework.^31^

### Identifying the research question

This scoping review aimed to answer the following question: what is the effectiveness of smoking cessation interventions for pregnant women in primary care and what factors contribute to this effectiveness? Based on this question, inclusion and exclusion criteria were determined.

### Identifying relevant studies

The following five electronic databases were searched: CINAHL, MEDLINE, Embase, Scopus, and Cochrane, using the key terms: (pregnancy OR pregnant OR prenatal OR antenatal OR perinatal OR maternal) AND (smoking OR tobacco OR cigarette OR nicotine) AND (general practice OR GP OR primary care OR primary healthcare OR family practice OR family medicine) AND (interventions OR strategies OR programme).

The inclusion criteria were as follows: studies involving smoking cessation interventions in pregnant women; studies undertaken in primary care settings (primary care, community clinics, or general practices explicitly stated); original research studies; and studies published in English.

The exclusion criteria were as follows: studies involving non-pregnant women; studies undertaken in secondary or tertiary care; non-original research; and studies not published in English.

### Study selection

The article titles yielded were screened manually by two authors and those determined as not eligible were eliminated. The abstracts of the remaining articles were screened and if considered relevant, the full article was reviewed.

### Charting the data

A data-charting spreadsheet adapted from the Joanna Briggs Institute (JBI) was developed. The following data were extracted: author(s); year of publication; country of origin; study setting; study design; study population and sample size; intervention details; control group; study outcomes; and key findings. Articles were analysed, producing a descriptive summary aligning with the aims of the review.

### Collating, summarising, and reporting the results

A narrative analysis of the heterogenous studies was completed and reviewed by three authors. The results were mapped and presented in accordance with the objectives. Gaps in knowledge were identified. Quality assessment of the evidence was not a primary objective.^31^ Following reporting of results, stakeholder input was sought. A primary care physician with expertise in addiction reviewed the results.

## Results

### Mapping the results

**Figure 1.**
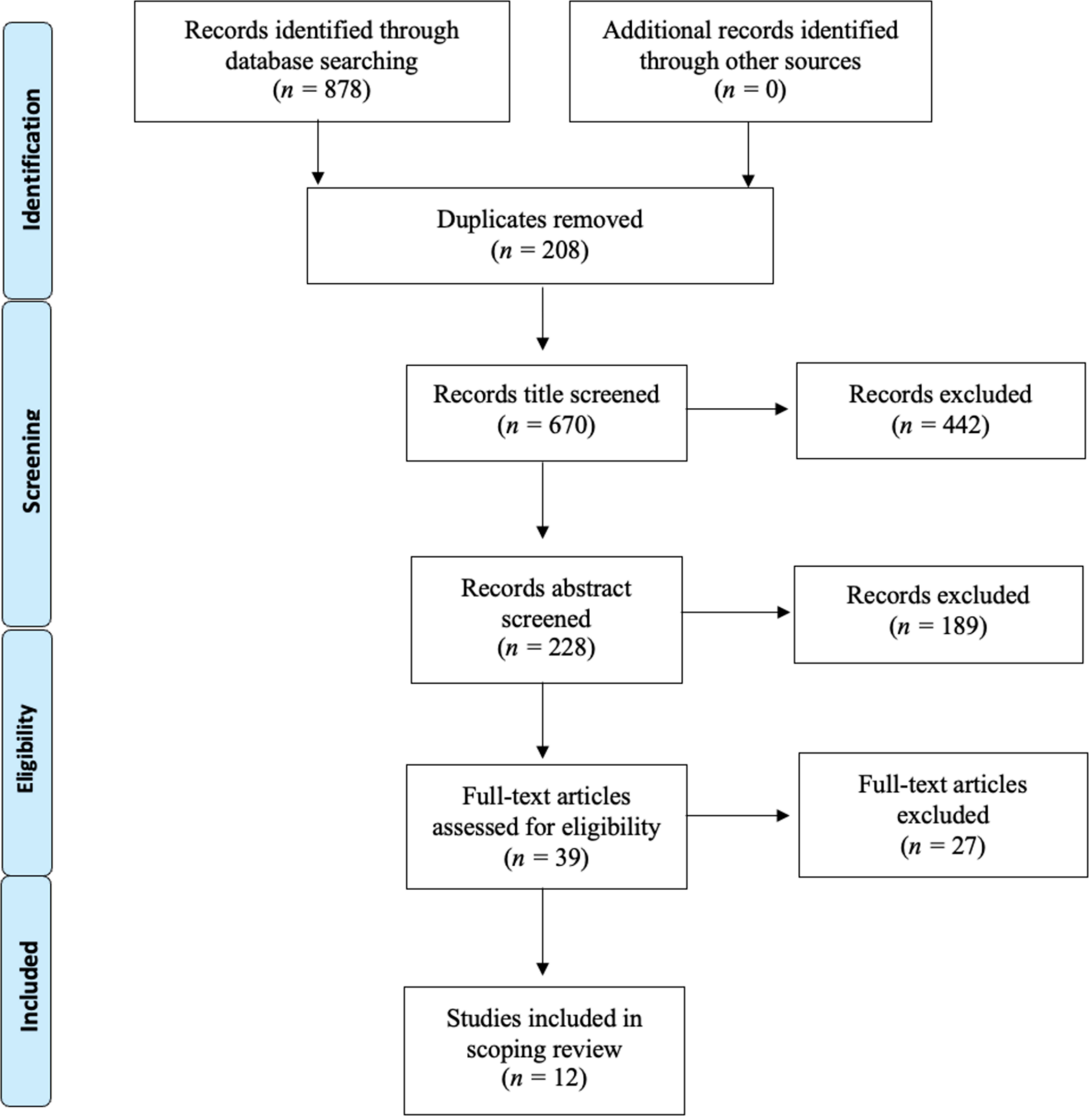
PRISMA flowchart

The database search yielded *n =* 878 articles. Following a duplicate screen, *n =* 208 articles were removed. Article titles were screened according to PRISMA guidelines and those deemed not appropriate (*n =* 442) were eliminated. The remaining *n =* 228 abstracts were reviewed according to the inclusion criteria. This resulted in *n =* 189 abstracts being eliminated and *n =* 39 full articles being reviewed. A further *n =* 27 articles were eliminated owing to the studies not fulfilling inclusion criteria. The final number of articles was *n =* 12 (Figure 1). The studies are summarised in Supplementary Table S1.

### Study characteristics

Study year ranged from 1989–2020, reflecting that smoking cessation in pregnancy is a long-standing issue in primary care. The range of countries comprised: the Netherlands (*n =* 1), Norway (*n =* 3), the UK (*n =* 2), the US (*n =* 3), Australia (*n =* 1), New Zealand (*n =* 1), and South Africa (*n =* 1). Methods used included randomised controlled trials (*n =* 5), non-randomised controlled trials (*n =* 2), cluster randomised trials (*n =* 2), prospective observational study (*n =* 1), quasi-experimental study (*n =* 1), and mixed-method study (*n =* 1). Study populations ranged from *n =* 109 to *n =* 7845. The stage of pregnancy at which participants were recruited varied: booking visit (*n =* 3), <20 weeks gestation (*n =* 1), <25 weeks gestation (*n =* 1), <28 weeks gestation (*n =* 3), 12–30 weeks gestation (*n =* 1), and unspecified gestational date for recruitment (*n =* 3).

### Study interventions

All interventions incorporated behavioural technique interventions. The exact intervention methods differed between studies but largely consisted of motivational interviewing, counselling, education, self-help, and financial incentives. The 5As intervention was used in two studies and one study focused on the transtheoretical (stages of change) model.^32–36^ NRT was offered in one study after two failed cessation attempts.^32^ The primary care facilities offering the intervention differed with *n =* 4 midwifery clinics, *n =* 3 general practices, *n =* 1 midwifery clinics and general practices, *n =* 2 primary care antenatal clinics, and *n =* 2 primary care antenatal clinics and general practices.

### Study outcomes

Five studies indicated that the intervention significantly reduced tobacco use in pregnancy.^37–41^ These interventions included the 5As intervention, psycho-educational methods, counselling sessions, motivational interviewing, and structured smoking cessation education. In two studies, where the intervention was worthwhile, participants were financially rewarded for participating in the study.^37,39^

Seven studies demonstrated the intervention did not cause a statistically significant increase in smoking cessation rates or reduction in the number of cigarettes smoked.^32–34,42–45^ Messimer *et al* reported that 28% of the intervention group quit smoking by 32–36 weeks gestation; however, this did not reach statistical significance.^45^ Lawrence *et al* reported that in two different intervention groups, 2.6% and 3.1% had sustained abstinence at 30 weeks gestation.^34^ Most of these seven studies reported positive results in intervention and control groups, indicating no significant effect from the intervention. In most studies, women in the control group received usual care from their primary care provider. Most studies described usual care as providing some smoking cessation advice, but not as intensive as the intervention.

Stakeholder consultation was not reported in any of the studies, demonstrating a lack of stakeholder involvement.

## Discussion

### Summary

This scoping review identified twelve studies that investigated the impact of smoking cessation interventions on pregnant women in primary care. The interventions may have increased smoking cessation rates in pregnant women; however, it cannot be determined if the increase was attributable to the interventions, or if pregnancy itself and increased contact with healthcare professionals were the reasons for decreased tobacco consumption.

### Strengths and limitations

Regarding strengths, this review strictly followed the methodology framework of Arksey and O’Malley.^31^ Following collation of results, stakeholder input was sought. A primary care physician with expertise in addiction reviewed and corroborated the results without identifying further gaps.

Regarding limitations, this study was limited to the population of currently pregnant women, and did not assess pre-pregnancy or post-partum periods. The heterogeneity of the study methods means that some studies contained information not specific to the research question. However, Arksey and O’Malley state that comprehensiveness is a critical component of scoping reviews.^31^ Studies not published in English were not reviewed, possibly introducing bias. It is also not possible to exclude selection and reporting bias in this study. Finally, this is a scoping review and therefore the quality of the studies included has not been assessed.

### Comparison with existing literature

Regarding effectiveness of smoking cessation interventions in primary care, most of the studies did not report a statistically significant difference in smoking cessation between intervention and control groups. However, it is important to note that even in the control group, some level of smoking cessation advice was offered. In all studies, there was some increase in smoking cessation rates or reduction in amount of tobacco smoked in both groups (if a control group was used). It is possible that interventions of any kind within primary care may positively impact smoking cessation or reduction rates. Pregnancy itself may be a strong motivator to quit smoking. One study reported that pregnancy was the indication most pregnant women stopped smoking, and the motivation could not be attributed to the intervention.^45^ Another study that found motivational interviewing and a financial incentive increased smoking cessation rates reported that women who enrol in cessation programmes may have higher motivational desire to quit smoking than those who do not enrol and therefore, it cannot be determined whether the interventions are completely responsible for increased cessation.^37^ Oude Wesselink *et al* showed the control group had the same cessation rate as the intervention group.^43^ From these results, it is difficult to attribute the cause of smoking cessation to a single factor.

Limitations were noted in some studies. Oude Wesselink *et al* found that not all recommended counselling steps were delivered.^43^ The cessation rate in women who received stage 1 of this counselling technique was 10%. This increased to 16% when all steps were completed. Regarding cost-effectiveness, Robling *et al* stated that adding Family Nurse Partnership (FNP) to usual care resulted in substantial additional cost, which was not substantiated by the intervention effects.^42^ In addition to the study by Robling *et al,* which investigated teenage mothers, only three other studies examined demographic and socioeconomic factors. One study involving the Aboriginal community determined that there was no additional benefit from a high-intensity quit-smoking intervention.^32^ Joseph *et al* investigated smoking among high-risk, pregnant African–American and Latina women and found that the intervention group more frequently resolved some or all of their risks.^39^ The low number of studies investigating demographic and socioeconomic factors related to this topic suggests that future research would be valuable.

Regarding different smoking cessation interventions, the interventions used in the five positive studies were as follows: motivational interviewing and a financial incentive; 5As intervention and psycho-educational methods; clinic-based individually tailored counselling sessions; structured smoking cessation education; and an information flip-over and booklet provided with extra GP consultations.^37–41^ The 5As intervention method used in another study and an intervention adapted from the clinical practice guideline *Treating Tobacco Use and Dependence* also resulted in significant reduction in smoking rates but was not deemed significant compared with the control group.^33,44^

Other interventions that were not clinically significant included home-visiting programmes, counselling, and a transtheoretical model based self-help manual. NRT was offered in one study but did not show a significant benefit for smoking cessation.^32^ It is not exactly clear why some of these interventions were effective while others were not but lack of training of healthcare professionals, poor delivery of the intervention, and pregnant women not participating as expected were reported as factors that limited the efficacy of interventions.

Regarding primary care settings and providers, McLeod *et al* acknowledged that most smoking cessation interventions for pregnant women have been studied in secondary or tertiary settings. This study found that in New Zealand, primary care midwives are increasingly expected to deliver health promotion messages to pregnant patients and often attend patients in their homes rather than clinics.^40^ Smoking cessation interventions may be more effective in this setting, although this has not been investigated. In a different New Zealand study, while midwives appear excellently placed to deliver brief interventions for smoking, only half of midwives reported offering interventions to pregnant smokers.^46^

Pregnant women encounter more difficulty in accessing smoking cessation services compared with non-pregnant women owing to potential stigma and insufficient programmes.^46^ One study illustrated the feasibility of non-medical professionals delivering primary care interventions and determined further research is required for identifying optimal methods to provide this service.^39^ Another study suggested that GPs should be trained further in this area.^41^ A range of healthcare professionals offered interventions in the five positive studies, including GPs, midwives, counsellors, and specially trained pregnancy advisers.^37–41^ There is scope for investigating interventions not provided by healthcare professionals such as those instigated by family and friends or through multimedia.

### Implications for research and practice

This scoping review allowed available literature to be mapped and summarised, and gaps to be identified. To understand factors that may strengthen or weaken motivation to cease smoking, further research is indicated. Pregnant women should be consulted for a collaborative approach and perspectives of primary care workers should be explored. Resource-directed aspects, such as cost-benefit analysis, would be beneficial. Investigating the relationship between factors, such as socioeconomic status and smoking cessation in pregnancy, would also be useful. The purpose of doing more research into this topic is to find appropriate interventions that can reduce smoking in pregnancy and therefore reduce harm to both mother and baby. Primary care is one of the most easily accessible healthcare areas as it is community based and it is often the first point of care for pregnant women. Therefore, it is an ideal area to provide smoking cessation interventions to this population.
